# Pollen counting made easy: mobile pollen counter provides real-time results in the field

**DOI:** 10.1093/jxb/erag047

**Published:** 2026-01-28

**Authors:** Benjamin S Lazarus, Viktoria C Wieser, Benjamin Fieber, Agnes S Dellinger

**Affiliations:** Department of Botany and Biodiversity Research, University of Vienna, 1030 Wien, Austria; Department of Botany and Biodiversity Research, University of Vienna, 1030 Wien, Austria; Department of Botany and Biodiversity Research, University of Vienna, 1030 Wien, Austria; Department of Botany and Biodiversity Research, University of Vienna, 1030 Wien, Austria; Ohio State University, USA

**Keywords:** Buzz pollination, pollen counting, pollen dosing, pollen release, real-time data acquisition, wind pollination

## Abstract

Understanding pollen release dynamics is essential for studying plant reproductive strategies, particularly in systems where pollen is aerosolized, such as wind- and buzz-pollinated flowers. Yet quantifying airborne pollen is often labor-intensive and dependent on laboratory-based methods, limiting field research. We demonstrate that a handheld air particle counter can provide rapid, portable, and precise quantification of pollen in real-time across pollination systems. Using controlled vibration experiments on buzz-pollinated *Melastomataceae* stamens, we compared counts from the handheld device with a conventional liquid particle counter. The handheld counter produced more consistent and realistic estimates, probably due to its ability to capture dispersed pollen clouds regardless of release direction. High-speed video confirmed that traditional methods can miss substantial pollen because of directional variability in complex stamens. We demonstrate that the device is applicable beyond buzz pollination by quantifying the exponential decline in airborne pollen concentration from wind-pollinated *Betula* anthers with increasing distance. The device also enabled fine-scale characterization of pollen size distributions and real-time release rates. The method proved robust to variations in pollen concentration and particle speed. Beyond pollen, this approach has potential for quantifying airborne particles such as spores, seeds, and pathogens, expanding opportunities for experimental field studies of airborne particle dispersal and plant reproductive evolution.

## Teaser

A handheld particle counter enables real-time, field-based quantification of aerosolized pollen, offering a fast, precise, and portable alternative to traditional, time-consuming laboratory-based pollen counting methods.

## Introduction

Pollen grains are the ubiquitous male reproductive agents of seed plants (gymnosperms and angiosperms) and share a unifying feature: dependence on abiotic (water, wind) or biotic (animals) vectors for transfer. Pollen release from reproductive structures (i.e. microsporangia in gymnosperms, anthers in angiosperms) is the critical first step in the pollen transfer process regardless of the pollen vector. Thus, plant traits which control pollen release, such as anther dehiscence, attachment, and biomechanical properties, as well as exposure of reproductive structures, are likely to be under strong selection (Timerman and Barrett, 2021), and may differ markedly depending on the efficiency of the pollen vector. It is commonly accepted, for example, that pollination by abiotic vectors such as wind is imprecise and inefficient, and is associated with traits facilitating the quick release of large amounts of pollen, while pollination by animals is commonly associated with more precise, efficient transfer and release of comparatively smaller pollen quantities (Linder, 1998; Ackerman, 2000; Friedman and Barrett, 2009). Even among animal-pollinated species, subtle differences in the rate of pollen release have been documented, with flowers adapted to more efficient pollinators such as hummingbirds releasing larger quantities of pollen per pollinator visit than flowers adapted to more wasteful (pollen-foraging) bee pollinators (Castellanos *et al*., 2006). Understanding how such variation in pollen release dynamics may serve to optimize the early steps of pollen movement would be highly important for more broadly understanding the evolution of plant reproductive strategies (Opedal *et al*., 2023; Štenc *et al*., 2023). To date, however, we lack large comparative assessments of pollen release dynamics, with just a handful of case studies available (Butcher *et al*., 2020a, b; Diaz-Martin *et al*., 2023; Kern *et al*., 2023).

Studying patterns of pollen release, such as how, when, how fast, and how much pollen is released from plant reproductive structures, requires the quantification of pollen grains, a task which remains laborious, and may explain the scarcity of studies comparatively or experimentally studying pollen release dynamics. Historically, the most common method for quantifying pollen of both zoophilous (Campbell, 1991; Adler and Irwin, 2006; Alonso *et al*., 2012; Koski *et al*., 2018; Larson *et al*., 2018; Pearson *et al*., 2023) and anemophilous plants (Jones *et al*., 2018) involved manually counting grains on a microscope slide or adhesive tape. Different approaches for gathering pollen have been developed in these research fields. In studies focusing on animal pollination, researchers have used fuchsin jelly (Kearns and Inouye, 1993) to capture pollen from the bodies of pollinators. For anemophilous species, various air samplers are employed, such as the gravimetric Durham, volumetric Burkhard, or impaction Rotorod configurations to pull pollen from the wind column (Heffer *et al*., 2005; Peel *et al*., 2014; Anderson *et al*., 2020). These approaches are time-consuming, require expertise to operate, and only return results once samples are processed in a laboratory. Furthermore, differences in the way that pollen is captured by each configuration can lead to variations in pollen counting accuracy as well as sensitivity to extrinsic variables such as wind speed, humidity, and pollen size (Heffer *et al*., 2005; Peel *et al*., 2014; Anderson *et al*., 2020).

More recently, a range of automated detection systems have been developed for anemophilous pollen (Buters *et al*., 2024) that attempt to either automate pollen identification from images (such as the BAA500, APS, Aerotrap, and ACPD) or use laser light scattering or fluorescence to identify pollen particles (such as the Poleno-Jupiter, Rapid-E, WIBS, IBAC-2, and KH3000). These methods provide higher temporal resolution and faster sampling rates but are all mounted devices that weigh ≥100 kg (including housing, Buters *et al*., 2024). de Weger *et al*. (2020) identified a need for a portable air pollen sampler with higher spatial resolution and developed their own ‘Pollensniffer’; however, this device still requires manual pollen counting under a microscope. In the peculiar case of buzz pollination, in which pollen particles are naturally aerosolized when bees (or researchers) apply vibrations to flowers, researchers have captured the ejected pollen streams in liquid-filled tubes (i.e. Eppendorf tubes) and then used liquid particle counters to quantify pollen (Dellinger *et al*., 2019, 2021; Kemp and Vallejo-Marín, 2021; Pereira Nunes and Vallejo-Marín, 2022; Vallejo-Marín *et al*., 2022). This process is user-friendly and faster than manual counting but requires a laboratory, many consumables, and several pre-processing steps such as sonication, homogenization, and subsampling/dilution. Furthermore, this approach can be prone to contamination from non-pollen particles and only provides results after samples are processed in the lab.

Quantifying pollen release dynamics is perhaps most important in two types of flowers: first, in wind-pollinated flowers, where large amounts of pollen may get lost in the environment if released at the wrong time (Rabinowitz *et al*., 1981; Keijzer *et al*., 1996; Timerman and Barrett, 2021), and second, in ‘pollen’ flowers, which offer pollen as the sole reward, so that opposing selective forces on pollen (functioning as reproductive agent and pollinator reward) probably balance pollen release rates to minimize pollen wastage (Vogel, 1978; Dellinger *et al*., 2019; Kemp and Vallejo-Marín, 2021; Heiling *et al*., 2023). Wind-pollinated and pollen-rewarding angiosperms have evolved repeatedly and constitute important agricultural crops (i.e. *Poaceae* such as wheat, barley, and maize; and buzz-pollinated crops such as tomatoes, eggplants, and blueberries) and sum up to ∼20% of angiosperm species (Buchmann, 1983; Ackerman, 2000; Cooley and Vallejo-Marín, 2021). However, their pollen release dynamics remain poorly explored. Showcasing tools and experimental approaches which allow for a fast, easy, and versatile quantification of pollen release from flowers with disparate morphologies and growing in diverse environments is essential, especially for establishing how external abiotic factors (i.e. wind, humidity, and temperature), floral biomechanical properties, and interactions with pollinators shape pollen release dynamics and hence plant reproductive success and fitness.

Here, we show how airborne particle counters can be used in botanical research as a versatile tool to quantify pollen release from flowers, dramatically reducing sample processing time and opening up avenues for assessing additional parameters, such as pollen release rates in real time. Using buzz-pollinated pollen flowers (*Melastomataceae*) as a model and employing vibration experiments with artificially synthesized vibrations to effect pollen release, we first compare traditional techniques of capturing pollen in Eppendorf tubes and pollen counting with liquid particle counters with real-time counts using airborne particle counters. Across species of different morphologies, we found pollen counts obtained with the airborne particle counter to be higher, and more precise, than with the traditional method. Employing video recordings, we show that the airborne particle counter can capture all released pollen grains, while substantial amounts of pollen grains are lost when capturing pollen in Eppendorf tubes. Airborne particle counters not only provide a more accurate method for quantifying pollen release but, given the immediate delivery of results through real-time counting, they also dramatically reduce sample processing time. Furthermore, we show that using airborne particle counters, we can obtain quick measurements of pollen grain size distributions and pollen release rates. Finally, using a wind-pollinated species (*Betula* sp.), we show that airborne particle counters are not limited to studying buzz-pollinated plants, and may be used to tackle questions related to pollen concentration decrease curves and pollen flight dynamics over distance. Thus, employing airborne particle counters may facilitate future research into pollen flow dynamics of plants with different pollination systems and morphologies through a reduction of processing time, increase in measurement accuracy, real-time measurements of pollen release rates, and versatility and ease to employ during fieldwork.

## Materials and methods

### Study system

We here use ‘pollen flowers’ from the plant family *Melastomataceae* to compare traditional liquid particle counting and a handheld airborne particle counter. The studied species exhibit traits typical for buzz-pollinated flowers (Dellinger *et al*., 2021): pollen as the only reward and restricted pollen release through longitudinally closed anthers only opening by a small apical pore (poricidal anthers). Bees are the only pollinators capable of producing vibrations in the range of 100–400 Hz required for releasing pollen from these anthers (De Luca and Vallejo-Marín, 2013; Vallejo-Marín, 2022). When bees land on *Melastomataceae* flowers, they grasp the stamens with their legs and mandibles and start vibrating their indirect flight muscles, thereby transmitting vibrations directly to the anthers, causing pollen ejection from the anther pores into the air. Pollen may then land on the body of the bee or other parts of the flower, or be lost into the air current.

For the purpose of this project, we used flowers of 11 species [*Arthrostemma ciliatum* Pav ex D.Don, *Chaetogastra* cf. *naudiniana* Decne., *Heterotis rotundifolia* (Sm.) Jacq.-Fél., *Medinilla loranthoides* Naudin, *Medinilla magnifica* Lindl., *Medinilla* sp., *Pleroma urvilleanum* (DC.) P.J.F.Guim. & Michelang., *Rhexia cubensis* Griseb., and *Centradenia floribunda* Planch.; May–August 2024] cultivated at the Botanical Garden of the University of Vienna, and flowers from species occurring in the wild, *Meriania speciosa* (Bonpl.) Naudin (Santa Maria, Boyacá, Colombia; October 2024) and *Pleroma angustifolium* (Naudin) Triana (Diamantina, Minas Gerais, Brazil; November 2024). These species represent different flower morphologies encountered among buzz-pollinated angiosperms and are hence suitable for a comparison of pollen quantification methods.

Wind-pollinated flowers also rely on pollen release through mechanical vibrations triggered by the motion of anthers in air currents (Timerman and Barrett, 2019, 2021). We thus also performed pollen counting experiments with a wind-pollinated birch species (*Betula* sp.) collected in the Botanical Garden of the University of Vienna (March 2024) to explore applicability of our airborne particle counter in anemophily.

### Handheld air particle counter

We used a ParticlesPlus 8506-30 handheld air particle counter (ParticlesPlus, Stoughton, MA, USA) which utilizes a vacuum pump to pull airborne particles into the device and then determines the particle count and size based on the scattering of light as the particles pass through a laser beam. Several other airborne particle counters are commercially available, but this model has a high maximum particle concentration (15 000 000 particles ft^–3^ or ∼530 000 000 particles m^–3^) and adjustable binning based on particle size (0.5–75 µm), covering the great majority of seed plant pollen sizes (Knight *et al*., 2010). The device has a flow rate of 2.83 l min^–1^, records temperature and humidity along with each measurement, and provides real-time particle count results during the measurement. Before each testing day, the device was cycled with the supplied particle filter attached for 60 s until no particles were detected. This ensures no particles were contaminating the interior of the device.

### Artificial vibration setup for buzz-pollinated pollen flowers

To date, most studies on pollen release in pollen flowers have been conducted in the genus *Solanum* (*Solanaceae*), which has a relatively conserved flower morphology with reflexed petals and anthers aggregated into an anther cone, releasing pollen in a straight jet downwards (Vallejo-Marín *et al*., 2022). Experimental protocols have been developed and employed across several *Solanum* species and species with similar morphologies (i.e. *Exacum* and *Cyclamen*) to study how vibrations affect pollen release (Nevard *et al*., 2021). Specifically, artificially synthesized vibrations may be applied to flowers to effect pollen release, with the simplest approaches using tuning forks (King and Buchmann, 1996) or electric toothbrushes (Konzmann *et al*., 2020; Tayal and Kariyat, 2021) and more refined setups allowing for modulation of vibration frequency and amplitude using manipulated vibration-transduction speakers as playback systems (Brito *et al*., 2020; Vallejo-Marín *et al*., 2022). In the latter, flowers are usually fixed in a narrow water tube, and vibrations are applied directly to stamens by grabbing anthers with fine tipped forceps attached to a metal rod fixed to the vibrating plate of the speaker (Brito *et al*., 2020). Pollen is then captured in an Eppendorf tube held beneath the anther cone. While this setup works well in flowers with the typical *Solanum* morphology, for more complex buzz-flower morphologies (i.e. variable stamen arrangement, Dellinger *et al*., 2021), modifications of the artificial vibration setup are required, since the variability in stamen arrangement may strongly affect the directionality and scatter of pollen release (Konzmann *et al*., 2020).

**Fig. 1.**
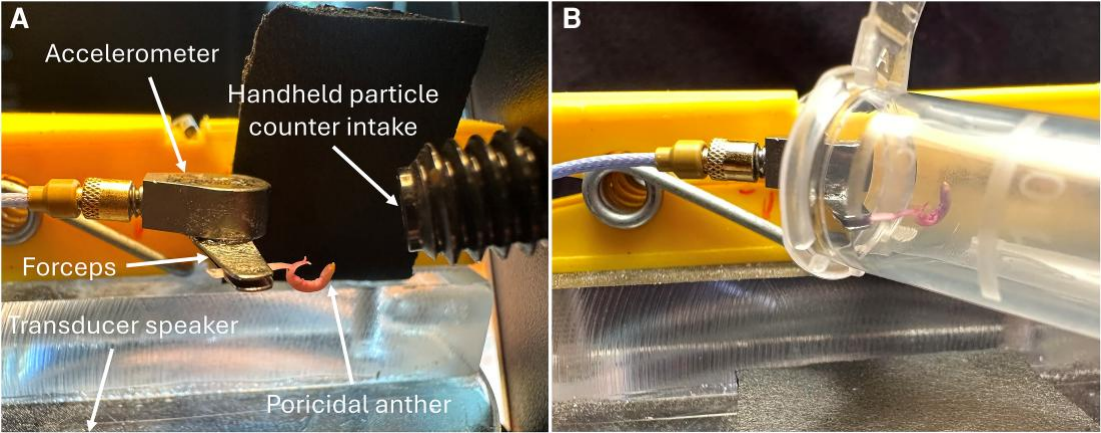
Experimental setups for artificial buzzing tests. (A) The handheld air particle counter and (B) the conventional pollen capture approach using a 2 ml Eppendorf tube.

For our experiments, we used Audacity v3.3.2 to synthesize pure 300 Hz sine waves (following Kemp and Vallejo-Marín, 2021) with each vibration lasting 5 s. We produced vibrations using a transducer speaker (Shenzhen Newadin Technology Co., Ltd, Shenzhen, China) with a clip mounted on a plexiglass plate attached to the top of the speaker. We removed a single stamen from a *Melastomataceae* flower, inserted the filament into the featherweight forceps, and inserted them into the clip. To monitor vibration amplitude (in m s^–2^) during playback experiments, we mounted a PCB model 352A21 accelerometer (PCB Piezoelctronics, Depew, NY, USA) on the forceps using superglue. During playback experiments, we quantified pollen release u sing either the traditional method (Eppendorf tubes and liquid particle counter) or the air particle counter (Fig. 1) to evaluate the accuracy of both methods across different vibration settings and applications.

### Validation: comparison of pollen counting techniques

For a baseline comparison between both measurement techniques (Fig. 1), we quantified pollen release from stamens of three species with varying morphologies (*Arthrostemma ciliatum n*=10 for each stamen type, *Chaetogastra* cf. *naudiniana n*=16, and *Medinilla* sp. *n*=16). Four stamens were randomly selected from each flower for testing. *Arthrostemma ciliatum* has dimorphic stamens so we ran separate tests on both stamen types (long and short). First, to quantify pollen release using the handheld particle counter, we placed the device directly in front of the pore during buzzing (Fig. 1A). We set the bin sizes to 0.5–10, 10–15, 15–20, 20–25, 25–30, and >30 µm, and considered the sum of particles larger than 10 µm (larger than pollen grain diameters in these species) as our overall pollen count. Prior to testing, we ran the particle counter in front of the setup without applying vibrations to the stamen to verify that the vacuum pump would not pull pollen grains out of the anther. Second, we performed identical buzzing tests but captured pollen grains in an Eppendorf tube by inserting the tip of the anther into the tube during vibration application (Fig. 1B, traditional method) (De Luca *et al*., 2013; Rosi-Denadai *et al*., 2020; Kemp and Vallejo-Marín, 2021; Pereira Nunes and Vallejo-Marín, 2022). We took care to ensure the Eppendorf tube did not touch either the stamen or the vibration system during testing. We then stored the captured pollen in 200 µl of 70% ethanol. After buzzing experiments were completed, we centrifuged this mixture and carefully removed the ethanol with a pipette. We added 1 ml of purified water to the tube and resuspended the pollen pellet by placing the tube in an ultrasound bath (10 min, 25 °C). We then pipetted 100 µl of the pollen solution into a liquid particle counter (Topas Particle Counter FAS362B), sorting particles into 62 size bins between 2 µm and 200 µm. Since errant particles that are the same size as pollen are inevitably present during liquid particle counting, pollen counts were determined as the sum of particles ±4 bins of the peak measurement within the range of 10–30 µm. This method captured the pollen peak that was not present when pure water samples were counted.

### Validation: exploring differences between counters using high-speed videos

To gain insight into why the two pollen counting methods may yield different results, we recorded high-speed footage of buzzing tests of five species with different stamen morphologies. These species compried *R. cubensis*, *M. magnifica*, *P. urvilleanum*, *H. rotundifolia*, and *C. floribunda.* The latter three species exhibit dimorphic stamens which we tested separately. We used a Sony Cyber-shot RX100 VII (Sony Group Corporation, Tokyo, Japan) to obtain high-speed (1000 fps) footage during buzzing.

### Validation: quantifying the effect of vibration velocity and pollen concentration on pollen count

Buzz-pollinated stamens can release particles at high speeds (0.25–2 m s^–1^) and in concentrated bursts, which may affect the counting accuracy of the air particle counter. It is possible, for example, that pollen grains that pass through the laser simultaneously or that are released in bursts may be detected as a single large particle (>30 µm) instead of the many smaller particles which they in fact are, thereby deflating count values. To evaluate whether high pollen release rates may confound pollen counting or sizing accuracy of the air particle counter, we tested whether increasing vibration velocity amplitude (leading to higher pollen release rates) leads to the detection of more oversized (>30 µm) particles. Comparing particle counts across varied vibration velocities further provides insight into the effect of particle speed on counting (i.e. are faster-moving particles harder to detect?). Hence, we performed buzzing experiments where we varied vibration amplitude continuously between 100 m s^–2^ and 350 m s^–2^ at 300 Hz and compared the number of particles detected and the proportion of particles in the larger bins. Due to constraints in flower availability, we ran tests on four new species (*n*>28/species): *H. rotundifolia*, *M. loranthoides*, *M. magnifica*, and *P. urvilleanum.*

### Application: pollen grain size measurements

The handheld air particle counter can detect particles with a diameter resolution of 0.45 µm. This enables the counter to filter non-pollen particles based on size. To verify the accuracy of the counter, we measured pollen grain diameters under a microscope (Olympus BX50, Tokyo, Japan) and compared these results with the particle sizes reported by the air particle counter. We used the same set of species as in the above experiment.

### Application: pollen release rates

Since the airborne particle counter provides real-time pollen counts, it may be used to study patterns in pollen release rates to understand, for example, whether stamens differing in morphology release pollen differently. To showcase this application, we extracted examples of fine temporal plots (during a 5 s test) of pollen release rates from the air particle counter. Note that the ParticlesPlus counter does not allow users to export temporal data directly, so that we extracted these curves visually using Webplotdigitizer (Rohatgi, 2011). We used two species, *M. speciosa* and *P. angustifolium*, for this experiment.

### Application: exploring pollen release dynamics in a wind-pollinated species

**Fig. 2.**
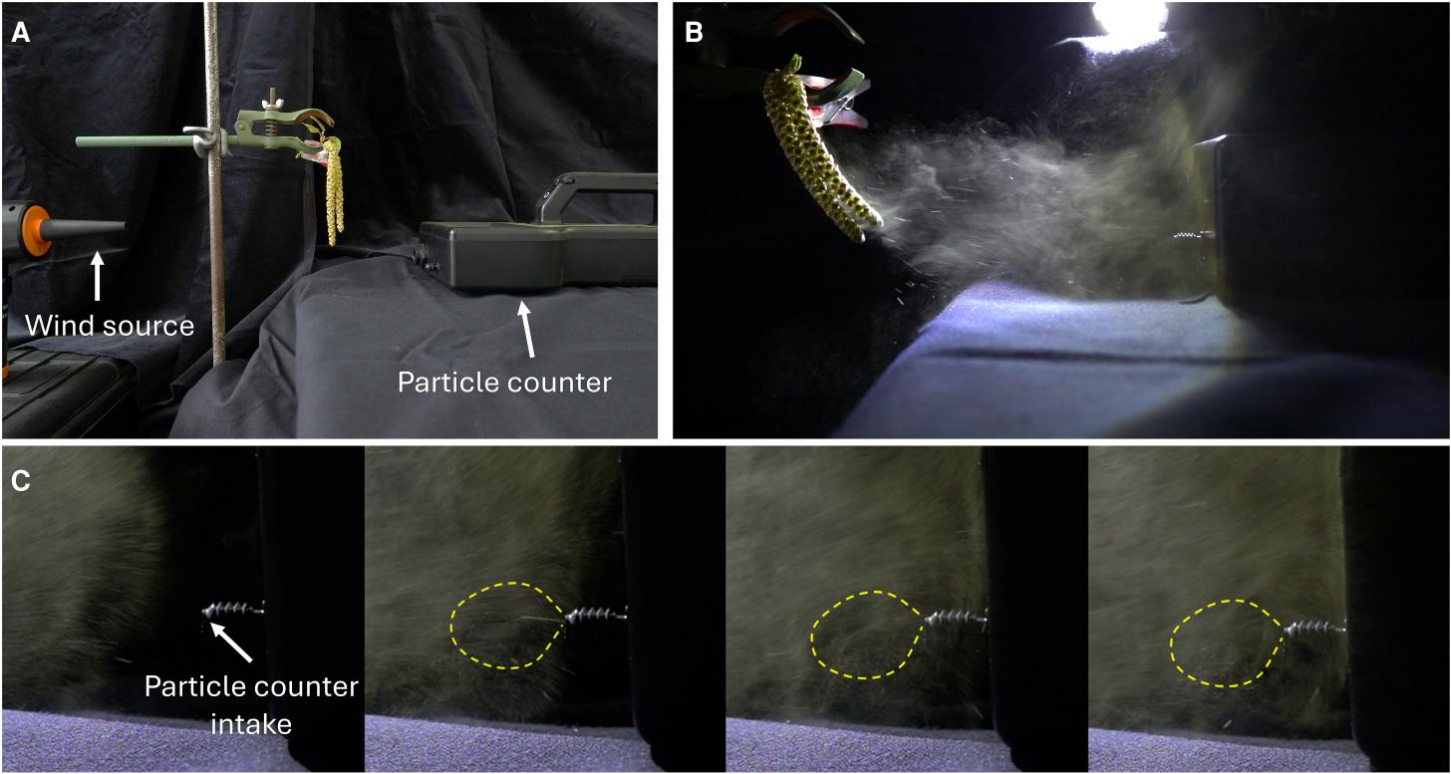
The air particle counter can also be applied to any pollination system which has aerosolized pollen, such as wind pollination. (A) Experimental setup for the wind pollination experiment using an artificial wind source applied to anthetic *Betula* sp. inflorescences and a particle counter to capture the released pollen. (B) Wind pollination simulation experiments showing pollen cloud being blown from the inflorescence towards the particle counter and (C) a time sequence showing the pollen cloud approaching the particle counter intake and the approximate intake volume where particles can be seen changing direction towards the counter vacuum.

To illustrate a potential use case for the air particle counter in wind pollination, we performed controlled experiments on birch flowers to measure how pollen density decreases with distance. We gathered closed (pre-anthetic) inflorescences from *Betula* sp. in the morning (∼09.00 h) and placed the peduncle in water. We kept the inflorescences in a closed cabinet until fully dehisced. We then chose clusters of two inflorescences of similar size (average length=62.2±11.1 mm) for this experiment (Fig. 2). Once anthetic, we transferred the inflorescences to a test stand by securing the peduncle using a clip. We used a handheld electric air duster (Whatook) to generate an air current, and a handheld anemometer (BT-100 wind gauge, BTMETER) to measure the wind current (5.1 m s^–2^) from a set distance of 20 mm. We exposed the anthetic inflorescences to this current for 10 s while placing the pollen counter at successively varying distances (10, 30, 50, 130, and 260 cm) from the inflorescences. We ran the counter for 5 s before and after the perturbations of the inflorescences. We also performed control runs of the same sampling time after every five tests to ensure no significant (<1 particle s^–1^) residual pollen was present in the air column. The pollen counter was placed at a height of 20 cm and the height of the inflorescences were adjusted so that when they were blown, the pollen cloud was directed at the counter intake (Fig. 2).

### Statistical analyses

We used a Shapiro–Wilk test alongside Q–Q plots to test for normality in buzz pollination experiments for each of the study species. Since the distributions measured by the liquid particle counter were not normally distributed, we performed Mann–Whitney U-tests to evaluate if pollen counts differed significantly between the two different counting techniques. We ran tests separately for each species. Since the results of wind pollination experiments were normally distributed within the counting distances, we conducted an ANOVA to determine if pollen counts varied significantly among different distance bins. We fitted an exponential concentration decrease curve with the form:

$$\mathrm{Pollen}\;\mathrm{count}=ae^{-b\times \mathrm{distance}(\mathrm{cm})}$$

to wind pollination simulation data in OriginLab 2025 following the results of Pluess *et al*. (2009) who found that this function best predicted paternity in anemophilous *Quercus lobata* Née. We determined fits by optimizing *a* and *b* to minimize the residual sum of squares.

To determine whether the detection of large (>30 µm) particles is more likely under high vibration velocities or pollen densities, we performed linear modeling in OriginLab 2025 using weighted least squares regression where vibration velocity (v) and total particle intake (c) were predictors and the number of large particles (>30 µm) was the response variable. We fit the data for each of the four species separately.

## Results

### Validation: comparison of pollen counting techniques

**Fig. 3.**
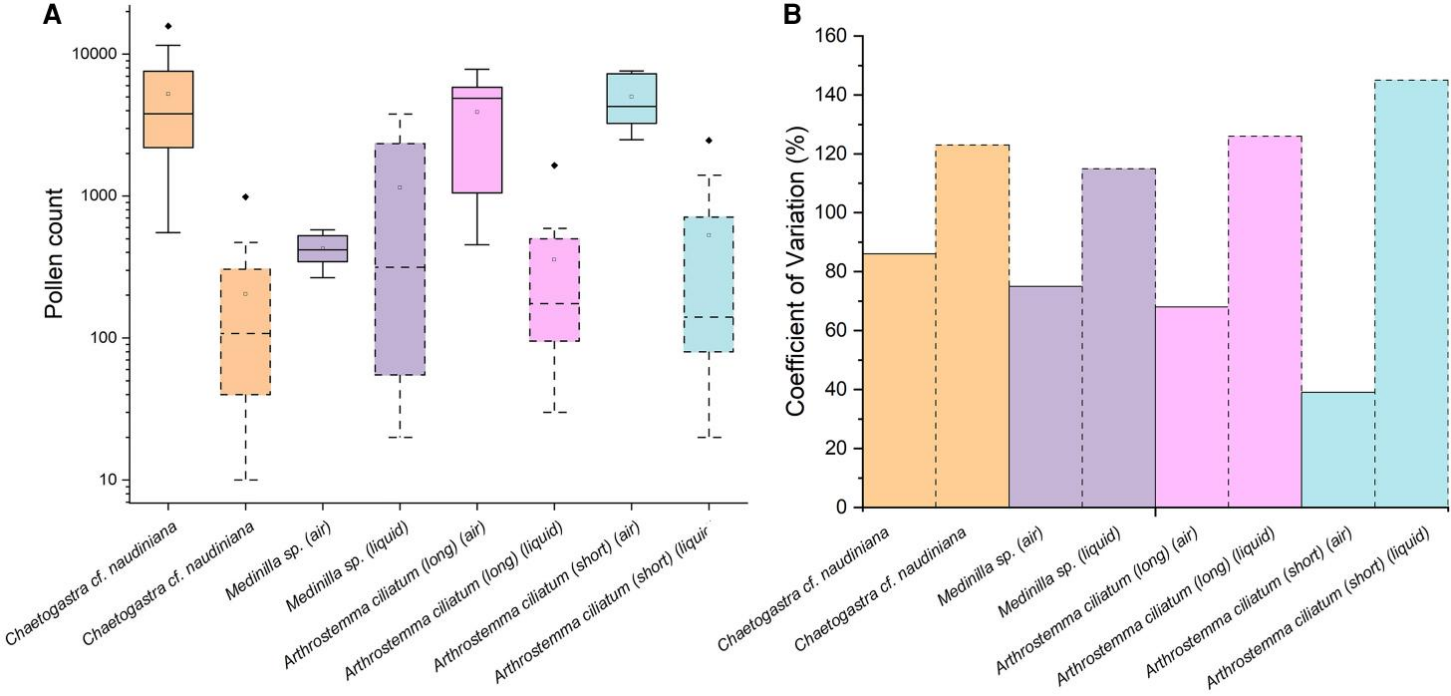
Results from artificial buzzing of poricidal stamens. (A) Box plots of pollen counts and (B) bar graphs of variation measured by both counting methods for each species.

The pollen release distributions measured by the air and liquid particle counting methods were significantly different for *Chaetogastra* cf. *naudiniana* (*Z*= −4.55, *P*<0.001) and the long (*Z*= −3.38, *P*<0.001) and short stamens of *A. cilliatum* (*Z*= −3.94, *P*<0.001), but not for *Medinilla* sp. (*Z*= −0.43, *P*=0.66) (Fig. 3A). The air particle counter measured a median pollen release that was 25–35 times higher for all species except *Medinilla* sp., which was only ∼2 times larger when measured with the air particle counter. The coefficient of variation of the pollen release distribution for all species was smaller when measured by the air particle counter (86, 75, 68, and 39, respectively) than when measured by the liquid particle counter (123, 115, 126, and 145, respectively) (Fig. 3B; Table 1). For all species, the results from the liquid particle counter varied significantly from a normal distribution according to a Shapiro–Wilk test while those measured by the air particle counter did not.

**Table 1.** The ratios of median values and coefficients of variation between the two tests for each species tested

	*Chaetogastra* cf. *naudiniana*	*Monochaetum* sp.	*Arthrostemma cilliatum* (long)	*Arthrostemma cilliatum* (short)
Ratio of median value (air/liquid)	35.4	1.9	27.9	30.7
Ratio of counter coefficient of variation (air/liquid)	70	65	54	27
Shapiro–Wilk test for normality *P*-value (liquid)	<0.001	0.0015	<0.001	<0.001
Shapiro–Wilk test for normality *P*-value (air)	0.073	0.055	0.27	0.12

**Fig. 4.**
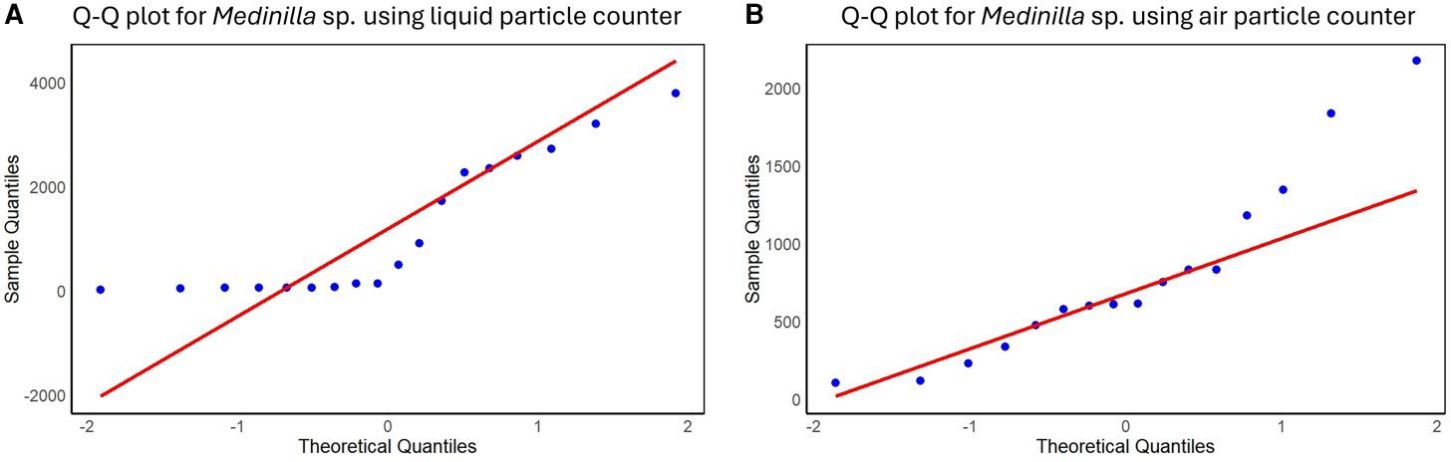
The air particle counter generally led to more normally distributed results than the liquid particle counter. Example Q–Q plots of results for artificial buzzing experiments on *Medinilla* sp. using (A) liquid and (B) air counters. The diagonal line in these Q–Q plots shows the expected points if the dataset was perfectly normally distributed. These plots suggest that the results for the liquid counter deviate more strongly from normality (and in a bimodal manner) than the air particle counter.

The bias of each type of counter is illustrated in Fig. 4 using *Medinilla* sp. as the example, which is the only species whose average pollen amount did not vary significantly between counter types. However, the distributions measured by each counter are different, with the liquid particle counter (Fig. 4A) indicating a bimodal distribution which strongly deviates from normality, while that of the air particle counter (Fig. 4B) is closer to normality but with a right skew.

### Validation: exploring differences between counters using high-speed videos

One possible explanation for the difference in pollen counts between counting methods is the way in which particles are captured (or not). The air particle counter uses a vacuum to pull particles out of the air which may help compensate for variable pollen release directions, meaning that pollen streams which are not initially directed towards the counter’s intake get pulled there (Video 1A). In contrast, pollen from a stamen of the same species, even when inserted nearly entirely into the Eppendorf tube, can escape capture (Video 1B). As a result, there may be more variability between tests using the traditional method due to high probabilities of pollen loss arising from stamen morphology and orientation. The bimodal distribution identified in the Q–Q plots for the liquid particle counter (Fig. 4B) may simply be a result of whether particles happen to be expelled into the tube or away from it.

### Validation: quantifying the effect of vibration velocity and pollen concentration on pollen grain count

While the relationship between total particle intake (c) and the number of particles >30 µm in diameter was significant (Table 2), the effect is not large, with *r*^2^ values generally <0.15. *Medinilla loranthoides* had an *r*^2^ of 0.2 but also had the fewest overall counts and very few counts over 30 µm. The velocity amplitude (v) of the vibrations had an insignificant effect on counts over 30 µm in two species and a negative effect in the other two, with similarly low *r*^2^ values. This suggests that neither the total number of particles nor their speed has a large impact on the number of oversized particles measured by the air particle counter.

**Table 2.** The fit equations for the percentage of pollen counts that are >30 μm based on the input vibration amplitude and the total particles counted

	*Heterotis rotundifolia*	*Medinilla loranthoides*	*Medinilla magnifica*	*Pleroma urvilleanum*
Total particle count (c)	Not significant	12.0c+10.7 *P*=0.017, *r*^2^=0.20	0.00064c+19.84 *P*=0.034, *r*^2^=0.08	0.0022c+40.5 *P*<0.001, *r*^2^=0.09
Vibration velocity amplitude (v)	−10v+3.9, *P*=0.0012, *r*^2^=0.12	Not significant	−384.6v+21.8, *P*=0.006, *r*^2^=0.13	Not significant

### Application: pollen grain size measurements

**Fig. 5.**
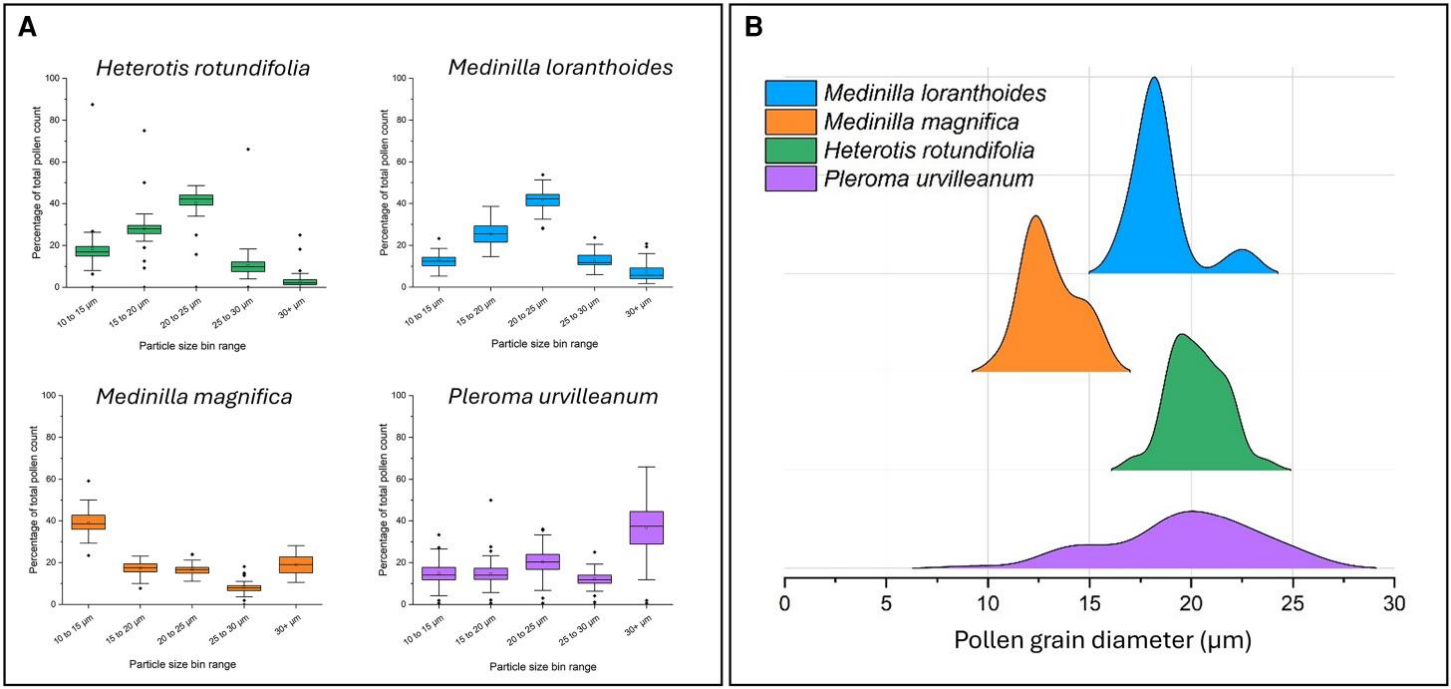
The air particle counter is also capable of accurately measuring pollen grain size. (A) Plots showing the percentage of the total particles detected in each particle size bin across species during artificial buzzing experiments and (B) pollen size distribution for different buzz-pollinated species measured under a microscope.

The percentage of particles counted in each bin (10–15, 15–20, 20–25, 25–30, and >30 µm) by the handheld air particle counter (Fig. 5A) aligned well with the relative frequency of pollen sizes for each species measured using a microscope (Fig. 5B). In all species, the relative quantity of large particles, which may occur due to pollen grains clustering as they pass through the device, was small, except for *P. urvilleanum*. However, in this species, we also observed a larger range of particle sizes and clusters of pollen grains under the microscope. Furthermore, this species released very few pollen grains during buzzing, perhaps due to clumps of pollen grains clogging the anther. In general, the air particle counter could accurately measure pollen sizes, and size binning clearly indicated the presence of pollen clusters. While quantifying pollen grains in such clusters is not possible, the ability to detect clusters may be useful since pollen clustering can play an important role in pollen transfer and may not be observed if particles are suspended in solution or homogenized with an ultrasound bath in preparation for a liquid particle counter.

### Application: pollen release rates

**Fig. 6.**
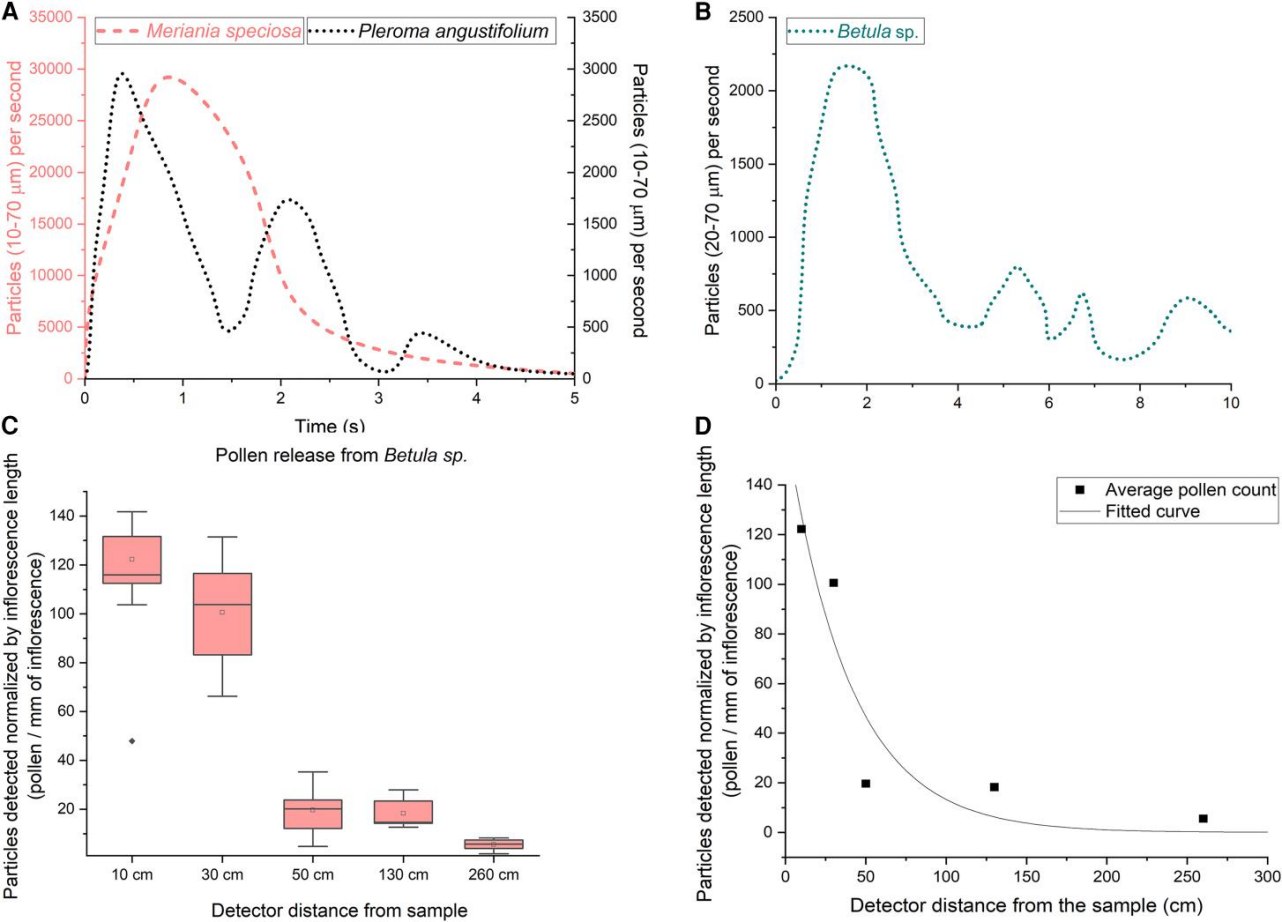
The quantity of pollen released across short time periods can also be measured using the air particle counter. Pollen particles detected per second for (A) two species of buzz-pollinated flowers during an artificial vibration test and (B) during wind pollination simulation tests of *Betula* sp. (C and D) Pollen release measured at different distances in artificial wind expulsion experiments including (C) box plots of pollen release at each distance and (D) a fitted concentration decrease curve alongside the average pollen counts measured at each distance.

The particle counter was able to detect differences in pollen release rate between two species with different stamen morphologies (Fig. 6A). *Meriania speciosa* with large, bulky stamens with smooth anther walls released most pollen in a single burst at ∼1 s, while *P. angustifolium* with narrow stamens with corrugated walls released pollen in three distinct bursts over 5 s. This temporal resolution of the particle counter can also be used to visualize the cyclical pollen release in anemophilous *Betula* sp. (Fig. 6B).

### Application: exploring pollen release in a wind-pollinated species

The amount of pollen of anemophilous *Betula* sp. detected by the particle counter varied significantly with distance (*F*=43.1, df=4, *P*<0.001) (Fig. 6C). We measured a sharp decrease in pollen counts when the detector was moved from 30 cm to 50 cm away from the pollen source. The exponential concentration decrease fit for pollen counts (Fig. 6D) with distance is

$$\mathrm{Pollen}\;\mathrm{count}=164e^{-0.025\times \mathrm{distance}(\mathrm{cm})}$$

This curve has a ‘half-life distance’ of 40 cm, meaning that only half of the initial quantity of pollen released should be detected at 40 cm.

## Discussion

To date, most studies on the number of pollen grains released from pollen flowers have been conducted in the genus *Solanum* (*Solanaceae*), which is amenable for experimental work given the comparative ease of cultivation. Many pollen flowers, including many *Melastomataceae*, are not easily cultivated, however, requiring the development of a portable pollen counting method that can be used in the field. Our results suggest not only that handheld air particle counters are a viable option for quantifying pollen release, but also that often they perform better than traditional benchtop liquid particle counters. For example, we measured a higher median pollen release and lower coefficient of variation for each species using the air particle counter relative to the liquid particle counter. In addition, air particle counters immediately deliver results, allowing for experimental adjustments on the fly. Liquid particle counters, in contrast, require laborious sample preparation, which itself has caveats and may introduce additional bias in pollen counts.

The liquid particle counting protocol includes a step to separate pollen clusters into individual grains using an ultrasound bath, which in turn should increase the total count of individual pollen grains. However, pollen counts from the airborne particle counter were significantly higher than those performed with the liquid particle counter, suggesting that the effect of clumping was relatively small. The handheld particle counter detected a substantial number of oversized particles (>30 µm), probably due to pollen grains clumping together, for only one species (*P. urvilleanum*) (Fig. 5A). However, this species only released a small quantity of total pollen which probably inflated the proportion of oversized particles. It is possible that other species have a higher proportion of clumped together pollen forming larger clusters. If this were the case, one would expect multiple peaks in different size bins or a tail in bins greater than twice the pollen grain diameter. In this instance, it might be necessary to ascertain the average number of pollen grains per cluster and the size of clusters on microscope slides in order to extrapolate the number of pollen grains represented by the counts in larger bins.

**Fig. 7.**
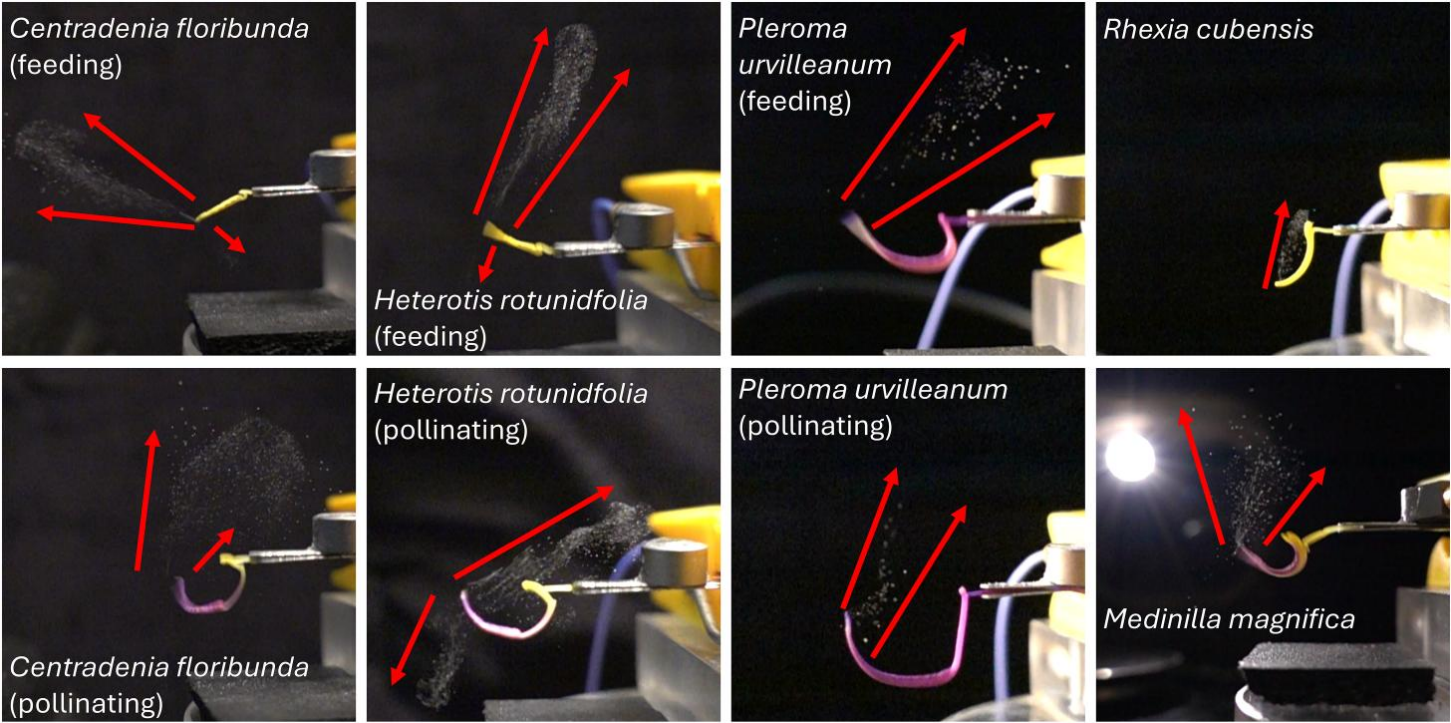
Pollen can be expelled by buzz-pollinated stamens in a variety of spatial patterns. These can vary between species and even within the different stamen types on the same flower. The red lines in this figure indicate the direction and approximate velocity of the pollen clouds expelled from stamens of different species vibrated with same the parameters (frequency=300 Hz, acceleration amplitude=300 m s^-2^).

In contrast to *Solanum*, where pollen is ejected in a predictable manner (usually downward) and can be captured accurately in Eppendorf tubes, more complex buzz-pollinated flower morphologies such as in *Melastomataceae* expel pollen in different directions (Fig. 7). Thus, capturing the entire pollen cloud in a tube for traditional counting can be challenging. Furthermore, different floral morphologies and perhaps even different vibration conditions can change the way that pollen is expelled (Amorim *et al*., 2017; Dellinger *et al*., 2019) making the direction of the pollen stream unpredictable. For example, in some *Melastomataceae* stamens pollen is expelled straight above the pore, yet the pollen cloud of others is expelled in a large arc, while yet others direct pollen back towards the filament (Fig. 7). In these more complex and varied stamens, the handheld particle counter may provide the added benefit of accurately capturing scattered pollen clouds. This variability in how pollen is expelled may partly explain why the pollen counts of certain types of stamens tested in this study varied more between the two methods. This variability also makes it difficult to ascertain a generalized conversion factor between the two methods.

Liquid particle counters also have the disadvantage of subsampling. The liquid particle counter used for this study, for example, can only count a maximum of 5000 particles ml^–1^. That means a sample of 0.2 ml with 10 000 pollen particles in it needs to be diluted to a 10th of the original liquid volume. This leads to extra transfer steps which can cause pollen loss and can lead to compounding errors that occur if the pollen is not homogenously distributed during counting. In addition, liquid particle counters detect a base level of noise caused by non-pollen particles that are present in liquid samples, even when using purified water. However, counting directly out of the air benefits from the fact that very few contaminants on the scale of pollen (>10 µm) are generally present in the air column. In control tests (*n*=10), where no buzzes were applied, the handheld air particle counter measured an average of just five particles in the same size range as pollen (Klein *et al*., 2006; Pluess *et al*., 2009).

Our tests on *Betula* sp. show that the handheld particle counter is not limited to buzz-pollinated species but can be used to measure, for example, pollen concentration decrease curves over distance in anemophilous species (Klein *et al*., 2006; Pluess *et al*., 2009). The fitted curve has relatively evenly distributed residuals (Supplementary Fig. S1) which suggests an appropriate fit. This implies that the handheld particle counter successfully quantified the well-established relationship between distance and pollen concentration, and can be extended to other studies of airborne pollen on short time scales.

### Studying pollen release dynamics in real time

Pollen release mechanisms have played a pivotal role in the evolution of seed plants. For example, successful wind pollination seems reliant on gradual pollen release when environmental conditions for dispersal are appropriate (Friedman and Barrett, 2009). In many habitats (e.g. grasslands, temperate, boreal, and montane forests), the most dominant plant species are wind pollinated (Regal, 1982). Researchers have used these distribution data to infer the macroenvironmental pressures (low rainfall, low habitat complexity, high windspeeds, and high temperatures) that drive the evolution of wind pollination (Whitehead, 1969; Culley *et al*., 2002; Friedman and Barrett, 2009; Rech *et al*., 2016). However, little experimental work has been done to explore how these abiotic factors truly affect, and how floral structures modulate, pollen release, leaving major knowledge gaps. Recent experimental work (Timerman and Barrett, 2018, 2019, 2021), for example, has challenged the paradigm that pollen would simply be pulled off dehisced anthers in wind-pollinated plants; instead, pollen seems to be released in a cyclical pattern in response to wind-induced vibrations (possibly due to resonance, which expels pollen periodically with each oscillation; compare our findings on *Betula* sp., Fig. 5B). Comparative experimental work on distantly related anemophilous plants such as *Poaceae* and Gymnosperms will be essential to establish generalities in how environmental conditions, floral traits, and pollen traits link together to modulate pollen release.

Similarly, researchers have proposed that the controlled, gradual release of pollen may be a driving strategy for the evolution of buzz-pollinated flowers (De Luca and Vallejo-Marín, 2013). Experimental work has shown that poricidal stamens of different species have variable dispensing rates which respond to buzzing conditions differently (King and Buchmann, 1995; Kemp and Vallejo-Marín, 2021). Air particle counters like the one presented here will allow a closer exploration of dispensing rates by tracking second-by-second pollen release, allowing for an improved understanding of how flower biomechanical properties modulate pollen release. Overall, rapid, real-time field experiments quantifying pollen release across populations, morphologies, and environments have the potential to disentangle hitherto under-appreciated functional complexities that occur when a pollination process involves aerosolized pollen.

### Experiments and quantification of small particles beyond pollen

Pollen is not the only small particle whose movement through air is of interest to research. The dispersal of spores, seeds, and pathogens often relies on air currents, and these small particles are hence subject to similar evolutionary pressures by the abiotic environment (Aylor, 1999, 2003; Brown and Hovmøller, 2002; Bouvet *et al*., 2006, 2007; Gralton *et al*., 2011). From an evolutionary perspective, the behavior of wind-dispersed seeds in various atmospheric and environmental conditions has long been an open question (Nathan *et al*., 2002). The size of wind-borne seeds varies across six orders of magnitude (Augspurger, 1986), with the smallest, near-microscopic diaspores presenting unique quantification challenges. Researchers have identified how both seed morphology (Zhu *et al*., 2022; Augspurger, 2024) and landscape conditions (Qin *et al*., 2022) play major roles in dispersal, yet most studies focus on macroscopic examples which are less prone to ‘floating’ on wind currents. Similarly, the spread of pathogens over both long (Brown and Hovmøller, 2002; Aylor, 2003; Gralton *et al*., 2011) and short distances (Aylor, 1999) often happens through the air, and understanding how airborne pathogen dispersal interacts with local landscapes (Bouvet *et al*., 2006, 2007) is important for protecting agricultural crops and human welfare. Easy-to-use, light-weight airborne particle counters like the one used here may open up new avenues for studying the evolution and functioning of particle release mechanisms and dispersal kernels, creating opportunities for identifying broad generalities in airborne particle movement across organismal units.

## Conclusion

Here we show that commercial handheld air particle counters can be a valuable tool in quantifying airborne pollen grains in the field. We found that in artificial vibration experiments, the air particle counter performed better (with more reasonable and precise results) than traditional benchtop liquid pollen counters. This is possibly due to the varied trajectories of pollen release from different stamens, which makes capturing pollen in an Eppendorf tube challenging. Meanwhile the vacuum function of the airborne particle counter inhales pollen moving in different directions, even away from the counter intake. We also show that this counting method can be extended to wind-pollinated taxa, can accurately measure pollen size despite high particle speeds or concentrations, and can provide valuable insights on pollen release data on a fine temporal scale (i.e. individual seconds). Moving forward, this approach also has the potential to be applied to other small, airborne particles such as spores and seeds to answer questions ranging from morphological adaptations of dispersibility to biogeographical evolutionary patterns.

Commercial handheld particle counters, like the one used in this study, provide several key benefits over existing pollen counting methods. For buzz pollination research, where pollen counting generally is a laborious, lab-based process, the handheld particle counter can be brought to the field, while also reducing processing time by providing immediate results. For field experiments, this can be a major boon that allows researchers to adjust experimental parameters during testing based on previous results. The handheld particle counter also uses fewer consumables (Eppendorf tubes and pipette tips) which can accumulate rapidly when generating large datasets with liquid particle counters. For wind pollination, the handheld particle counter is lightweight and mobile, unlike many existing particle counters, which opens up new experimental possibilities. The handheld particle counter can also be used on either short or longer time scales (10 h of continuous run time or indefinitely if plugged in). For both buzz and wind pollination experiments, the device also measures relative humidity and temperature, which have a significant impact on aerosolized particle movement, and can be factored into detailed analyses.

## Supplementary Material

erag047_Supplementary_Data

## Data Availability

All relevant datasets have been deposited in the public repository Phaidra: https://phaidra.univie.ac.at/o:2192131. A protocol for using the handheld air particle counter can be found at dx.doi.org/10.17504/protocols.io.x54v9b2mpl3e/v1.

## Supplementary data

The following supplementary data are available at *JXB* online.

Fig. S1. Q–Q plot of residuals for the decay curve fitted to the measured pollen release for *Betula* sp. artificial wind pollen release tests.
