# Supplementary material for: Pollen counting made easy: mobile pollen counter provides real-time results in the field
Source: J Exp Bot. 2026 Jan 28;77(10):2901–13. doi: 10.1093/jxb/erag047 (PMC13270332; doi:10.1093/jxb/erag047)

Supplemental information

Q-Q plot of residuals for decay curve fitted to *Betula sp.* pollen release

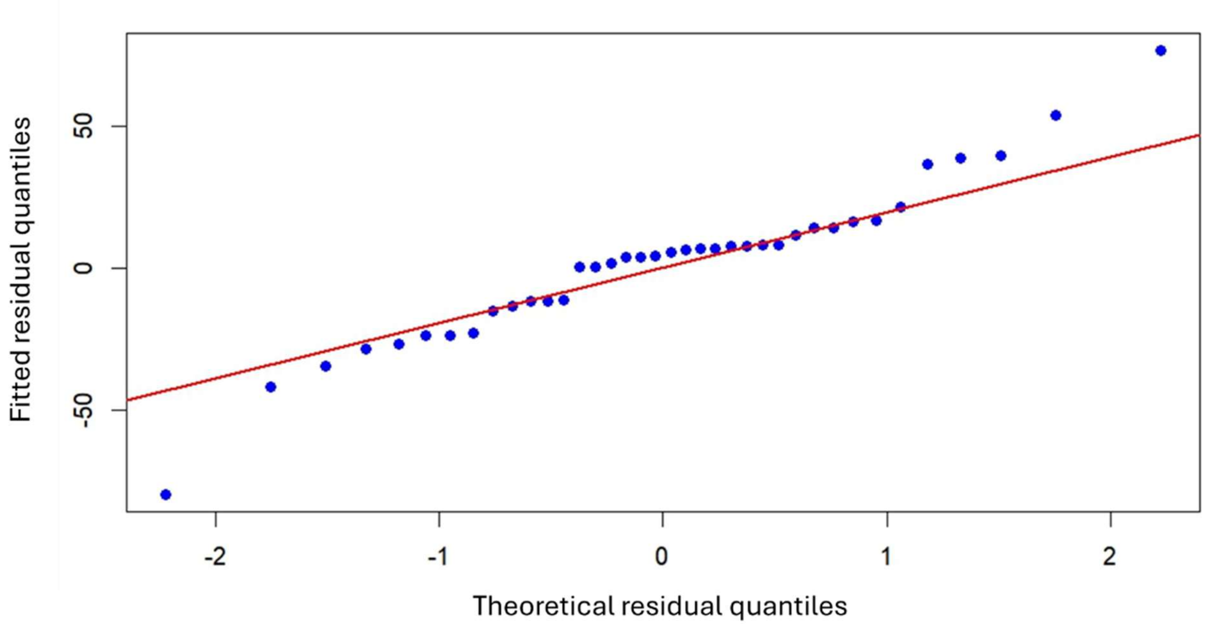

Supplement: erag047_Supplementary_Data [file erag047_Supplementary_Data.pdf]
